# Histopathological Variability and Concomitant Lesions in Pterygium in a Large Case Series

**DOI:** 10.1155/2021/6623794

**Published:** 2021-03-19

**Authors:** Sabrina Bergeron, Hiroaki Ito, Yves E. Dossous, Miguel N. Burnier

**Affiliations:** ^1^The MUHC–McGill University Ocular Pathology & Translational Research Laboratory, McGill University, Montreal, Quebec, Canada; ^2^Diagnostic Pathology, Graduate School of Medicine, Kyoto University, Kyoto, Japan; ^3^Department of Ophthalmology, McGill University, Montreal, Quebec, Canada

## Abstract

Pterygium is a common lesion consisting of fleshy conjunctival growth extending towards the cornea. There is no documented risk of malignant transformation; however, concomitant disease is not rare, and its link to sunlight exposure indicates a risk of other malignancies. The purpose of our study is to describe histopathological features of resected pterygiums and to recognize patients at risk of other conjunctival diseases. One hundred and forty-nine formalin-fixed and paraffin-embedded pterygium samples were subjected to histopathological analysis. Histological H&E sections were obtained and digitalized using a Zeiss Axio Scan.Z1 slide scanner. Thirteen predefined morphological features were used to record histopathological changes in the epithelium and substantia propria. Neovascularization was observed in 54% of the samples. Sun damage, comprising solar elastosis and stromal plaque, was present in 81% of the samples. Variation in epithelial thickness was the most common change, with acanthosis and atrophy being observed in 62% and 26% of the samples, respectively. In our series, 21% (31/149) of pterygiums showed mild to moderate dysplasia, a finding that may be associated to ocular surface squamous neoplasia (OSSN). Moreover, 32% (47/149) of the cases showed melanocytic hyperplasia, which could represent primary acquired melanosis (PAM). There is a positive correlation between dysplasia and chronic inflammation (*p*=0.012) and an inverse correlation with epithelial atrophy (*p*=0.001) and neovascularization (*p*=0.05). Similarly, a positive correlation is observed between goblet cell hyperplasia and melanocytic hyperplasia (*p*=0.02). Our findings show that pterygiums harbour histological features that may be suggestive of OSSN or PAM in 53% of our patients. Whilst being on the benign side of the spectrum, these two entities are known for their potential progression to malignancy. A recommendation is made for all surgically excised pterygiums to be sent for histopathological diagnosis, and clear guidelines for reporting of these lesions should be established. Associated histopathological findings suggestive of other concomitant diseases should be identified to insure adequate follow-up of these patients.

## 1. Introduction

Pterygium is a superficial growth of loose connective tissue that is vascularized and covered by an epithelium of varying thickness. For many years, and still today, this is an accepted histopathological description for pterygium. To this definition, some may also add prominent elastotic degeneration of the stroma and variable inflammatory infiltrate, all of which is often accompanied by “consistent with clinical diagnosis of pterygium.”

The issue with this, far too generic, histopathological description is that it does not tap into the deceptive side of the pterygium, the one where preneoplastic growth and atypia may be hiding. In fact, a pterygium is often much more than a fleshy overgrowth and may sometimes indicate underlying processes leading to more serious pathologies.

An accurate description would resemble the one from Chui et al.: “a centripetal growth of a leading edge of altered limbal epithelial cells followed by squamous metaplastic epithelium with goblet cell hyperplasia, an underlying stroma of activated fibroblasts, neovascularization, inflammation, and extracellular matrix remodelling.” [1] The presence of epithelial atypia and melanocytic hyperplasia may possibly be added to this description, and then one can have a complete assessment of pterygium.

The potential of pterygium to hide other, premalignant lesions has been widely discussed over the past decade. Its ability to grow blood vessels and invade adjacent corneal tissue is closely resembling neoplastic processes. Notwithstanding that recurrence rate is highly variable and may reach up to 78% in some instances [2–4], yet, these lesions are still often approached as stromal degenerative disorders and treated conservatively. With growing evidence pointing towards a preneoplastic process, a shift in the clinical management of pterygia is foreseeable.

Clinically, pterygium presents as a fleshy conjunctival growth at the limbus and extending towards the cornea. Its etiology is unknown; however, its prevalence in populations living near the equator suggests a link to ultraviolet exposure. The term “pterygium belt” was coined to describe regions saddling both sides of the equator where pterygium is highly prevalent, affecting up to half of the population in some areas [2].

The symptoms associated with pterygium may range from mild discomfort, foreign body sensation, and itchiness to blurry vision, depending on the extent of the growth. In many cases, pterygium does not require treatment; however, a surgical excision may be indicated for larger lesions where vision is hindered or for cosmetic reasons.

While surgical treatment, with or without adjuvant, is often sufficient to relieve symptoms, it is estimated that the recurrence rate is often very high and unpredictable [4]. Risk factors for recurrence are unclear; however, it is believed that pterygium is caused by a combination of sun, wind, and dust. All three factors need to be considered to prevent recurrences; hence, this condition is sometimes referred to as “surfer's eye.”

Based on histopathological findings from a subset of “atypical” pterygiums, a careful histopathological review of all excised lesions is recommended. Moreover, as demonstrated by the growing number of case reports in the literature where presumed pterygiums were found to be more serious, neoplastic processes advocate for a careful histopathological assessment of all pterygiums [5–10].

As microscopic examination is the gold standard to rule out neoplasia, many groups have attempted to characterize histopathological findings for pterygium [1, 11–19], and no definite consensus has been made on how to report on pterygium.

While most studies are from rural cohorts located within the “pterygium belt” geographical area, we conducted a 13-point systematic histopathological review of 149 excised pterygiums in a large city centre in Canada. Our assessment includes epithelial and stromal changes in each specimen and their possible link to other ocular surface diseases, such as ocular surface squamous neoplasia (OSSN) and primary acquired melanosis (PAM).

In agreement with current opinions, our result shows histopathological similarities between excised pterygiums and other premalignant ocular surface diseases. Henceforth, we recommend a thorough evaluation of all excised pterygiums and hope for a consensus on reporting of these lesions. We also recommend a close follow-up of these patients, regardless of whether they live in a high-risk geographical area or not.

## 2. Methods

This is a retrospective study. Out of a total of 854 conjunctival biopsies received in our laboratory between the years 2012 and 2019, 235 were diagnosed as pterygium. The mean age of patients at the time of the surgery was 56 years old, and there was a slight predominance of males (3 : 2 ratio).

One hundred and forty-nine formalin-fixed paraffin-embedded pterygium samples were randomly selected and subjected to histopathological analysis by a postdoctoral research fellow in ocular pathology (SB). Histological H&E sections were obtained and digitalized using a Zeiss Axio Scan.Z1 slide scanner. Morphological changes in the epithelium and substantia propria were recorded according to thirteen predefined morphological features listed in Table 1.

## 3. Results

The normal conjunctival tissue is composed of nonkeratinized stratified columnar epithelium overlying loose connective tissue. The epithelium thickness is variable between 2 and 5 rows of cells, and it also comprises goblet cells that secrete mucin, generating moisture for the ocular surface. The underlying stroma is called substantia propria and is a host for the immune system's mucosa-associated lymphoid tissue (MALT). For each of the 149 pterygium samples, one H&E section was digitalized and initially screened to confirm the quality of the selected tissue slide. Based on our observations and on previous studies [1, 12, 15], we proceeded to score each sample according to predetermined histological features. The microscopic features were then sorted according to their location within the conjunctival biopsy, epithelium or substantia propria. These histopathological findings are listed in Table 1.

Neovascularization, or the formation of new vascular channels, is reported in 54% (*n* = 81) of the analyzed samples. Histopathologically, the newly formed vessels have a plump-appearing endothelium, which may sometimes be leaky and leading to hemorrhage (observed in 32% of samples). In view of pterygium as a proliferative disorder related to sun exposure, the formation of new vessels, along with solar elastosis and stromal plaques, is a hallmark feature of pterygium.

**Figure 1:**
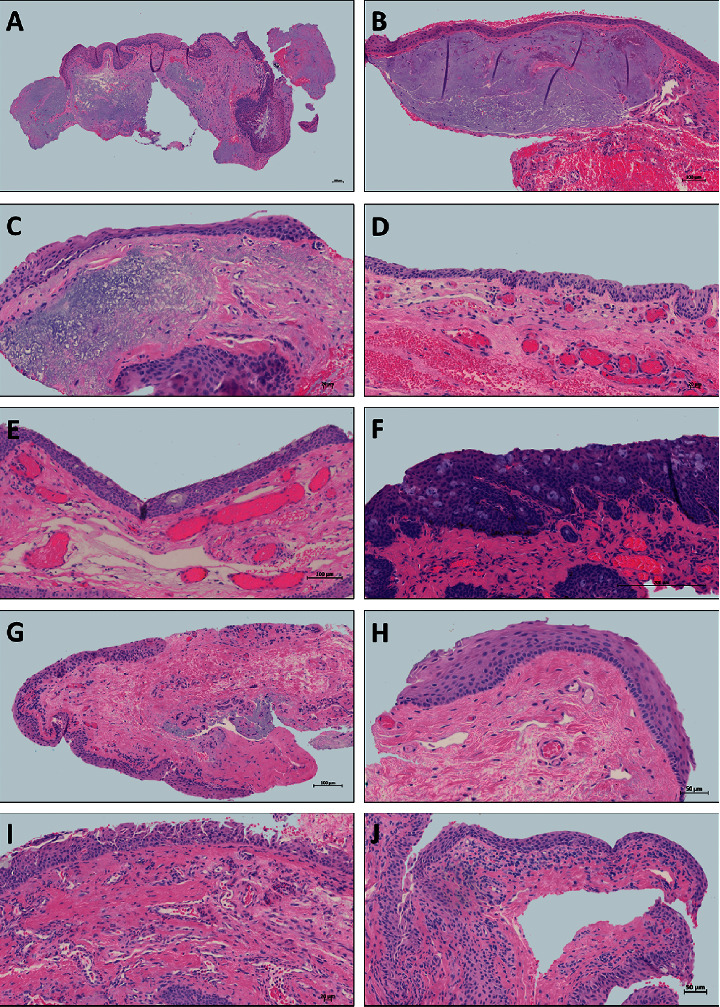
Representative images of the pterygium. (a) Low-power image showing the epithelium of varying thickness, hemorrhagic areas, and pronounced solar elastosis. (b) Stromal plaques underneath thinning epithelium. (c) Epithelial acanthosis and atrophy pronounced solar elastosis forming plaques. (d) Neovascularization and hemorrhage, mild solar elastosis. (e) Goblet cell hyperplasia with acanthosis, neovascularization, and mild solar elastosis. (f) Goblet cell hyperplasia, melanocytic hyperplasia, acanthosis, neovascularization, and lymphocytic infiltrate. (g) Basal cell hyperplasia, neovascularization, and solar elastosis. (h) Basilar hyperplasia, neovascularization, and mild elastosis. (i) Acanthosis and acute and chronic inflammation composed of neutrophils, eosinophils, histiocytes, and lymphocytes. (j) Lymphoplasmacytic infiltrates and histiocytes in a mucinous stroma.

**Figure 2:**
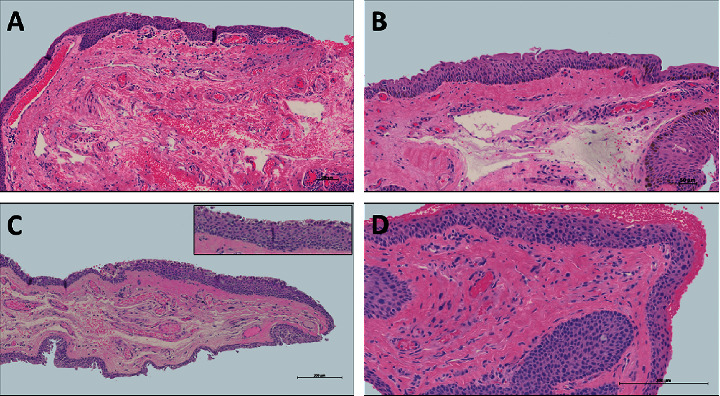
Abnormal histopathological features in a pterygium. (a) Melanocytic hyperplasia without atypia, neovascularization, lymphocytic infiltrate, and solar elastosis. (b) Melanocytic hyperplasia without atypia and neovascularization. (c) Epithelial atypia, neovascularization, area of epithelial atrophy, and mild hemorrhage (inset: higher magnification of epithelial atypia). (d) Moderate atypia and solar elastosis.

Solar elastosis is described as the elastotic degeneration of the collagen fibers within the substantia propria, and it is observed in 35% of the cases (Figures 1(a), 1(c)–1(e), 1(g), and 1(h) and Figures 2(a) and 2(d)). As elastosis progresses, we observe the formation of stromal plaques, consisting of homogenous, bland, eosinophilic subepithelial plaques (Figures 1(b) and 1(c)). The formation of plaques is observed in 46% of cases, and it is believed to be linked to the longstanding pterygium. There is a significant correlation (Pearson's *r* = −0.680; *p* < 0.01) between the presence of plaques and stromal elastosis. As both findings are thought to be linked to UV exposure of the conjunctival tissue, they are put under one umbrella feature called “sun damage.” This predominant feature is present in 81% of our samples.

The most commonly recorded histological changes in the conjunctival transitional epithelium are variations in epithelial thickness, acanthosis (thickening) or atrophy (thinning), observed in 62% and 26% of cases, respectively (acanthosis: Figures 1(a), 1(c), 1(e), 1(f), and 1(i) and atrophy: Figures 1(b) and 2(c)). The observation of both acanthosis and atrophy in the same specimen is not uncommon, and it is represented in 5.3% (*n* = 8) of our study samples. Hyperplasia of the basal cells is defined as small clusters or general “crowding” of the basal layer of the epithelium. The hyperplastic basal epithelium generally consists of small cells with high nuclear-to-cytoplasmic ratio as depicted in Figures 1(g) and 1(h). This characteristic is observed in 28% (*n* = 41) of our samples. Immunohistochemistry to verify the origin of these cells was not performed; however, it is believed that these clusters are of stem cell origin [1], which will be later discussed. Similarly, hyperplasia of goblet cells is observed in 5% (*n* = 7) of the cases (Figures 1(e) and 1(f)).

Other findings including the degree of inflammation and the nature of the inflammatory cells present may be attributed to the chronicity and extent of the lesion at the time it was excised; most samples are displaying chronic lymphoplasmacytic infiltrates (*n* = 33) and histiocytes (*n* = 38). Only a few samples show predominantly acute inflammation composed of neutrophils (*n* = 4).

Histological changes in pterygium samples that could be linked to other, potentially premalignant, pathologies are also frequently reported. In our series, 21% (*n* = 31) of pterygiums showed mild to moderate epithelial atypia (Figures 2(c) and 2(d)), a finding possibly associated to early ocular surface squamous neoplasia (OSSN).

An epithelium is considered to be atypical when the orientation and arrangement of cells is disrupted or when nuclear changes are observed. Atypia can be reactive or dysplastic (i.e., in response to inflammation or signaling a preneoplastic process). Out of 31 pterygiums presenting with atypia, twelve samples also show dense lymphoplasmacytic infiltrates (possibly reactive atypia), and 19 are likely to be truly dysplastic. The grade of atypia depends on whether or not the full thickness of the epithelium is involved. As shown in Figures 2(c) and 2(d)), the stratification of the epithelium is disrupted, and the cells are not showing a normal maturation pattern. Cells are often pleomorphic and may also show mitotic activity (not shown). Severe atypia was not observed in any of the samples.

In our study samples, 32% (*n* = 47) of pterygiums showed melanocytic hyperplasia (Figures 2(a) and 2(b)), which could represent primary acquired melanosis (PAM). If the basal layer of the epithelium shows a higher density of melanocytes than usual, it is considered as melanocytic hyperplasia (Figures 2(a) and 2(b)). None of these samples showed atypia.

The results show a positive correlation between atypia and chronic inflammation (*p*=0.01) and an inverse correlation with epithelial atrophy (*p*=0.001) and neovascularization (*p*=0.05). Similarly, a positive correlation is observed between goblet cell hyperplasia and melanocytic hyperplasia (*p*=0.02). No correlation is observed between epithelial atypia and melanocytic hyperplasia (*p*=0.44).

## 4. Discussion

### 4.1. Histopathological Findings of Pterygiums

Our results show that the histopathological review of excised pterygiums may benefit to identify unsuspected diseases. In line with other similar reports, histopathological analysis from 149 samples reveals cellular features that are apparent to OSSN or PAM in 53% of cases. Whilst most reports focus on either squamous lesions or melanocytic lesions, our group performed a thorough histopathological review assessing for both squamous and melanocytic histopathological features in the same study sample.

A large study reviewing 3,021 histopathological reports of conjunctival lesions found that the frequency of unsuspected OSSN within pterygium is 0.65% [11]. In a second study performed in Canada, the reported rate of OSSN is 2.33% [20]. As both these percentages are based on a search through histopathological reports, we suspect this number to be an underrepresentation of the reality as not all excised pterygiums are sent for histopathological review, and, when they are, there is no consensus on reporting of these lesions.

Consequently, a histopathological review of 533 excised pterygiums performed by a group in Australia reports an incidental finding of OSSN in 9.3% of the lesions, with 9.6% of them bearing severe atypia and one case of squamous cell carcinoma [12]. Similarly, our results show atypical epithelium with mild or moderate alterations in 21% of the cases. While no malignant lesion was observed in our sample, we cannot exclude the possibility of a progression to malignancy in these cases should they have not been excised.

While the numbers for OSSN-like features in pterygium are impressive, the presence of melanocytic hyperplasia in many pterygium samples should not be overlooked. A histopathological analysis from Perra et al. reported melanocytic hyperplasia that was otherwise undetected during clinical examination in nine of the eighty analyzed pterygiums, totaling 11% of the study samples [15]. Out of the nine, two lesions had melanocytic hyperplasia with atypia. Similarly, our results show that almost one-third (32%) of the studied cases show some extent of melanocytic hyperplasia. While no premalignant or atypical lesions were recognized from our study, the sole presence of the pigment in these samples warrants a thorough histopathological analysis of the specimen in order to rule out any atypical melanocytic proliferation (PAM with atypia or melanoma in situ).

Chui et al. conducted an analysis of 100 pterygium samples to assess for squamous and melanocytic changes in the same sample [1]. Their result showed that five cases had pterygium concomitant with OSSN and seven cases with a PAM or nevus. There is no indication whether these findings are observed in the same lesions or not. Based on our result, 21% of cases showed epithelial atypia, and 32% showed melanocytic hyperplasia. The Pearson correlation coefficient (*r* = −0.063) between both features is nonsignificant (*p*=0.44), hence suggesting two different subsets of patients at risk for “abnormal” pterygium.

An explanation for the discrepancies in percentages across all studies may be related to geographical location, inside or outside of the pterygium belt. It may also be linked to the hospital or clinic where the patient is treated. In practices where pterygium is treated with a conservative approach, surgical excision may be reserved only for certain types of lesions. Additionally, since there are no specific guidelines for histopathological reporting, the scoring of epithelial atypia depends on the quantity and availability of the tissue, as well as the observers' personal tolerance for atypia.

It is worth noting that some atypical epithelial cells may also be observed in the specimen showing a pronounced inflammatory reaction (reactive atypia); these changes are not true dysplastic changes. In our study, we observed a significant positive correlation between atypia and chronic inflammation, signaling that perhaps some of the atypia is reactive. However, in 19 out of 31 cases with atypia, there was no notable inflammation, suggesting a true dysplasia in at least 13% of our study samples.

While most histopathological studies on pterygium are in agreement with our findings, two reports with conflicting results were found during our literature review. The first is from Israel, within the “pterygium belt,” and the second is from Toronto, an area of similar latitude as ours [16, 18]. Both studies reviewed 682 and 1127 pterygiums, respectively, and identified no signs of OSSN in their samples. The discrepancies may be attributed to the sampling methodology, where only case reports of confirmed pterygium were reviewed. Nonetheless, both authors agree that excised pterygiums should still be submitted for histopathological examination to rule out other diseases.

Confronted to an abundance of evidence supporting histopathological atypia in excised pterygiums, we ought to question ourselves regarding the true definition of a pterygium. Are these findings the result of two concomitant diseases or are they part of a larger spectrum of diseases? In order to prove the latter, a histopathological review of confirmed OSSN and PAM should be performed in an attempt to identify microscopic features of pterygium in these specimens.

### 4.2. Cellular Origins of Pterygiums and Disease Classification

For many years, pterygiums were viewed as degenerative diseases of the conjunctival stroma. Even though pterygium etiology is not yet fully understood, there is substantial evidence pointing towards a disease of proliferative nature. Our observations suggest that pterygiums are highly vascularized lesions that show signs of growth, both in the epithelium and substantia propria, therefore, supporting the hypothesis of a proliferative disease. We observe newly formed vascular channels in over half of our samples, epithelial basilar hyperplasia in 28% of the studied pterygiums, and epithelial atypia in 21% of them.

A previous report from an Australian group suggested that cell clusters at the origin of epithelial basilar hyperplasia are in fact stem cells with proliferative potential (CK15, CK19, and p63 positive) [1]. The group proposed these aggregates to be the origin of the typical Fuchs flecks that are clinically observable during the slit-lamp examination. It is hypothesized that these clusters are kept in an “inactive” state until an unknown event takes place and activates the proliferation of this “invasive front,” leading to the progression of the pterygium. Moreover, it had been shown in a separate study that pterygium tended to have a higher proliferative index, as demonstrated by Ki-67 immunohistochemistry, than normal conjunctival samples [14].

The concept of a premalignant lesion has been around for many decades. In fact, Hogan and Zimmerman described pterygium with nuclear atypia as “active keratosis.” [21] Moreover, Clear et al. described pterygium (solar keratosis) as part of a spectrum of diseases that may lead to carcinomatous changes [22]. Over fifty years have passed, our understanding of pterygium has evolved, yet it is still far too often viewed as an uneventful degenerative change of the cornea. As it was brilliantly described by Chui et al., pterygium may be viewed as “stem cell disorders with premalignant features.” [1]

In an attempt to find common grounds with pterygium and other ocular surface diseases, UV exposure appears to be an obvious choice. In fact, ophthalmohelioses is a term coined to designate all ocular diseases that are thought to be directly related to sun exposure. Our study may be based in Canada, far away from the so-called “pterygium belt”; however, ophthalmohelioses are not infrequent. A study by Coronero suggested that even in regions further from the equator, such as Greenland, ultraviolet exposure may be similar to equatorial regions if ground reflectivity is taken into account [23]. Given that Canada is famous for its winter activities, the reflectivity of UVB rays even during the cold winter months may pose a risk for UV-related pathologies. In populations where the resources are available, each of these lesions should be assessed carefully to avoid complications for the patients and to minimize the burden on healthcare costs in cases of disease progression.

### 4.3. Histopathological Diagnosis of Pterygiums

As we, research groups specialized in ophthalmic pathology, value histopathological examination of all excised pterygiums, it is our duty to identify and report these atypical lesions in order to insure a proper follow-up of these patients. There is an unmet need for the establishment of guidelines for processing and reporting pterygium samples in ophthalmic pathology; it is imperative that the specimen is correctly oriented and processed in order to visualize the lesion in its entity, and then histopathological reports should address all epithelial and stromal findings. Each report should also indicate which cellular findings are typical of pterygium and which may indicate underlying benign or premalignant processes.

Our 13-point list for histopathological analysis of pterygium was inspired from previous studies [1, 12, 15, 19] and adjusted based on our findings. We recommend the use of a similar list for the histopathological diagnosis of pterygium in order to coin any cellular change that could reveal an underlying condition. Aside from accurate histopathological diagnosis and improved patient care, the use of a diagnostic checklist merged with clinical data will allow research groups to identify possible subsets of patients that are more at risk for pterygium associated with OSSN and/or PAM.

In the management of pterygium, other UV-related pathologies such as skin cancer should be accounted for patients presenting with ophthalmohelioses. As suggested by an extensive Taiwanese study, the risk of pterygium is 2.15 times greater in patients who had previous diagnosis of nonmelanoma skin cancers [17].

Additional findings such as histopathological findings between primary lesions and recurrent ones, as well as other relevant ocular and clinical history (HPV infection, immune status, and transplant), should also be documented in an attempt to pinpoint which lesions are more at risk of complications or which ones will likely carry an uneventful course of the disease.

As OCT imaging of ocular surface is gaining in popularity, we encourage the use of noninvasive imaging modalities to assess conjunctival lesions prior to excision. While OCT does not replace histopathological analysis of the lesion, its use to distinguish pterygium from OSSN has shown to be a great addition to the clinical management of these, sometimes, challenging lesions [24–27].

## 5. Conclusion

In conclusion, we reviewed 149 pterygium samples and proceeded to a thorough histopathological analysis. Our results point out that possible early signs of ocular surface squamous neoplasia or primary acquired melanosis were identified in 53% of our patients. These findings point out to a time for change in the management of pterygiums. We recommend that pathologists and ophthalmologists from large healthcare centres work together to establish a standardized sampling and reporting methodology for pterygiums.

## Figures and Tables

**Table 1:** Histopathological characteristics of the pterygium.

—	—	Number of cases	% of cases
Epithelium	Acanthosis (thickening)	93	62
	Atrophy	39	26
	Hyperplasia of goblet cells	7	5
	Hyperplasia of basal cells	41	28
	Hyperplasia of melanocytes	47	31.5
	Dysplasia (mild or moderate)	31	20.8

Substantia propria	Solar elastosis	52	35
	Stromal plaques	69	46
	Sun damage (elastosis + plaques)	121	81
	Neovascularisation	81	54
	Hemorrheage	47	32
	Acute inflammation (neutrophils)	4	3
	Chronic inflammation (lymphoplasmacytic infiltrate)	33	22
	Histiocytes	38	26

## Data Availability

The datasets used and/or analyzed during the current study are available from the corresponding author upon reasonable request.
